# Citicoline in Ophthalmological Neurodegenerative Disease: A Comprehensive Review

**DOI:** 10.3390/ph14030281

**Published:** 2021-03-20

**Authors:** Francesco Oddone, Luca Rossetti, Mariacristina Parravano, Diego Sbardella, Massimo Coletta, Lucia Ziccardi, Gloria Roberti, Carmela Carnevale, Dario Romano, Gianluca Manni, Vincenzo Parisi

**Affiliations:** 1 IRCCS-Fondazione Bietti, Via Livenza, 3, 00198 Rome, Italy; oddonef@gmail.com (F.O.); diego.sbardella@fondazionebietti.it (D.S.); luxzic@hotmail.com (L.Z.); gloriaroberti82@gmail.com (G.R.); carmela.carnevale@fondazionebietti.it (C.C.); vmparisi@gmail.com (V.P.); 2 Eye Clinic, ASST Santi Paolo e Carlo, San Paolo Hospital, University of Milan, Via Antonio di Rudinì, 8, 20142 Milan, Italy; luca.rossetti@unimi.it (L.R.); romanodario@gmail.com (D.R.); 3 Department of Clinical Sciences and Translational Medicine, University of Rome Tor Vergata, Viale Oxford 81, 00133 Rome, Italy; coletta@uniroma2.it (M.C.); gianlucamanni53@gmail.com (G.M.)

**Keywords:** retinal ganglion cells, citicoline, apoptosis, proteasome, neuroprotection, neurodegeneration, glaucoma, ischemic optic neuropathy, diabetic retinopathy

## Abstract

Cytidine 5’-diphosphocholine has been widely studied in systemic neurodegenerative diseases, like Alzheimer’s disease, Parkinson’s disease, and brain ischemia. The rationale for the use of citicoline in ophthalmological neurodegenerative diseases, including glaucoma, anterior ischemic optic neuropathy, and diabetic retinopathy, is founded on its multifactorial mechanism of action and the involvement in several metabolic pathways, including phospholipid homeostasis, mitochondrial dynamics, as well as cholinergic and dopaminergic transmission, all being involved in the complexity of the visual transmission. This narrative review is aimed at reporting both pre-clinical data regarding the involvement of citicoline in such metabolic pathways (including new insights about its role in the intracellular proteostasis through an interaction with the proteasome) and its effects on clinical psychophysical, electrophysiological, and morphological outcomes following its use in ophthalmological neurodegenerative diseases (including the results of the most recent prospective randomized clinical trials).

## 1. Introduction

Retinal ganglion cells (RGCs) bodies reside in the ganglion cell layer of the inner retina and their axons run above them in the retinal nerve fiber layer to constitute the optic nerve, which brings visual input to the cerebral structure [1].

RGCs may be affected in several pathologies of the visual system, like glaucoma, optic neuritis, and diabetic retinopathy.

Open Angle Glaucoma (OAG) is characterized by a loss of all RGCs compartments: somata, axons, and dendrites. It has been described that several pathogenetic pathways underly RGC death in glaucoma including chronic intermittent ischemia, redox imbalance, excitoxicity, defective axon transport, trophic factor withdrawal, autophagy dysregulation, and accumulation of amyloid beta [2,3,4]. 

Moreover, several studies have demonstrated that neuroinflammatory response is strongly involved in glaucoma; the inflammatory pathway can contribute to RGCs loss, due to several stressors, such as, among others, the aberrant purinergic signaling with P2X7 receptor (P2X7R) activation [5].

Similar mechanisms of RGCs death are involved in optic neuritis, which, together with glaucoma, are the major degenerative causes of optic nerve damage. 

In anterior ischemic optic neuropathy (AION), an irreversible, painless, and acute vascular failure of the optic nerve, cell death cascade is due to many factors, such as an increase of intracellular calcium, excitotoxicity, the generation of free radicals, blood-retinal barrier disruption, inflammation, and apoptosis [6].

In diabetic retinopathy (DR), neurodegeneration is the primary event, and it may precede the development of the characteristic microvascular complications [7]. The targets of this neurodegenerative process are the RGCs and amacrine cells with consequent structural thinning of retinal neuronal and axonal layers at macula [8]. The downregulation and loss of neuroprotective factors, the increase of oxidative stress and lipid peroxidation, the excitotoxicity induced by glutamate, and the dysregulation of pro-inflammatory factors are the main pathogenic mechanisms that are implicated in this process [9].

Given the essential role of neurodegeneration in the pathogenesis of these diseases (OAG, AION, and DR), it is reasonable to hypothesize that therapeutic strategies that are based on neuroprotection may be effective in slowing down RGCs loss.

Among the molecules with neuroprotective effect, citicoline, also known as Cytidine 5’-diphosphocholine, has been widely studied in systemic neurodegenerative diseases, like Alzheimer’s disease (AD), Parkinson’s disease, and brain ischemia [10]. It is interesting to highlight that, among the common characteristics of AD and Glaucoma, is amyloid beta deposition in the brain and in the retina of AD and glaucoma patients, respectively. Moreover, it has recently been discovered that specific miRNAs are biomarkers and therapeutic targets, in glaucoma and other neurodegenerative diseases, such as AD [11].

Since its very first discovery and usage in preclinical models and in clinics, citicoline has showed a multifactorial mechanism of action linked to phospholipid homeostasis, redox homeostasis, mitochondrial dynamics, cholinergic and dopaminergic neurotransmission [12,13,14]. As a matter of fact, citicoline displays an identical chemical composition and structure to that of endogenous CDP-choline, a precursor of glycerophospholipid phosphatidylcholine, which is naturally synthesized in living cells, starting from choline and cytidine, through an enzymatic biosynthetic pathway [15,16]. Together with phosphatidylethanolamine, phosphatidylcholine is an essential phospholipid for the maintenance of intracellular and extracellular membranes of eukaryotic organisms; these molecules are of particular relevance in surveying neuron homeostasis and functionality, by serving membranes turnover, synaptic plasticity, and neurotransmission [17,18,19]. 

Furthermore, citicoline stimulates the biosynthesis of sphingomyelin, a key lipidic metabolite that contributes to the stabilization of plasma membrane of RGC axons. This property further helps the cells to inhibit the release of free fatty acids and it confers additional protection toward redox imbalance, through improved scavenging properties, and to-ward the release of neuroinflammation modulators [15]. 

Upon oral intake, citicoline is thought to be rapidly absorbed and then hydrolyzed to choline and cytidine in the intestinal wall and liver; therefore, besides providing the metabolic precursors of phospholipids, as cited above, enters synthetic pathways of nucleic acids, proteins, and acetylcholine [20]. 

Citicoline is envisaged to confer a multifaceted protection towards neurodegeneration by reducing glutamate excitotoxicity and oxidative stress, increasing neurotrophin levels, enhancing the release of neurotransmitters (such as norepinephrine and dopamine), improving axonal transport deficits and supporting axon regeneration, promoting mitochondrial function, and modulating insulin signaling [15,21]. In addition, citicoline prevents ischemia-induced tissue accumulation of free fatty acids and reduces infarct volume and brain edema, thus resulting in an antiapoptotic effect that is related to mitochondrial dependent cell death mechanism [22,23]. 

Nowadays, citicoline is available as intra-muscular injection oral formulation and eye-drops.

Preclinical data will be discussed in this review as the rationale for the use of citicoline in human pathologies involving RGCs and clinical studies (reporting psychophysical, morphological, and electrophysiological evidences) that validated the effect of citicoline in ophthalmological neurodegenerative diseases, like OAG, AION, and DR. 

## 2. Rationale for the Use of Citicoline in Ophthalmological Neurodegenerative Diseases: Pre-Clinical Data

Despite the proven therapeutic efficacy, which is widely discussed throughout this text, citicoline pharmacology still lacks solid molecular and biological evidences that support its mechanism(s) of action in vitro and in vivo [16,24].

As far as phospholipids, choline and dopamine, which can be synthesized starting from citicoline metabolites, are macromolecules of key relevance to neuron life, and this biosynthetic-restricted hypothesis on drug activity does not convincingly account for the broad neuroprotective activity that the drug is recognized to display in vitro and in vivo [16,25]. Several cellular and mammalian experimental models of brain and retina pathologies, spanning from neurodegeneration, excitotoxicity, metabolic insult, and vascular damage, further discussed below, have raised several concerns regarding the pharmacology of this drug and have long suggested the existence of alternative mechanisms of action of the intact molecule or of the metabolites that it is made of (Table 1).

First, citicoline pharmacokinetic and pharmacodynamic properties do not clarify whether citicoline actually is the active compound, a prodrug, or, more likely, both [16,22,25]. Citicoline has been long thought to rapidly undergo hydrolysis and, probably, dephosphorylation by plasma phosphatases into choline and cytidine and used to de novo synthesize the endogenous CDP-choline, as introduced in the previous paragraph [16,25].

If that is the case, then it is unclear why: (i) upon citicoline absorption, the choline concentration in blood is much lower and not equimolar to that of cytidine; (ii) citicoline is several-fold less toxic in animals than choline, when administered at the same dose, which implies that the choline moiety released upon drug hydrolysis represents only a minor fraction or else it enters, for unknown reasons, into a catabolic pathway more quickly than pure choline does. 

Furthermore, the administration of the drug following the topical application of citicoline 2% ophthalmic solution has recently allowed for detecting the intact drug, together with its metabolites, at a high concentration in the humor vitreous of human subjects, implying that neuroprotection can also likely be achieved in human subjects also with the intact molecule in vivo [26]. 

Most notably, although citicoline displays a limited stability over time when dissolved in common laboratory buffers (unpublished observation), the robust biological effect, which has been reported in independent experimental settings in vitro (devoid of the blood passage and drug hydrolysis), indeed supports the existence of specific activities of the intact molecule.

As anticipated before, citicoline has shown very significant efficacy in vitro and in vivo in experimental models of vascular, metabolic excitotoxic, and neurodegenerative conditions. 

Citicoline administration to rat primary retinal cell cultures conferred protection from apoptosis, by means of a reduced frequency of caspases activation and accumulation of apoptosis markers, in the presence of glutamate-induced excitotoxicity and high glucose challenge [27]. 

Remarkably, protection against glutamate toxicity had been previously related to the upregulation of glutamate transporters, such as EAAT2 in lipid rafts, in a stroke experimental model [28]. 

Regarding excitotoxicity, the intraperitoneally injection of citicoline prevented the pathological thickening of retinal layers in a rat experimental model of kainic acid (KA)-induced toxicity [29]. In this experimental setting, drug delivery significantly attenuated the extent of cell death, induced intravitreal administration of the glutamate analogue, and further rescued the choline acetyl-transferase and tyrosine hydroxylase immunohistochemical pattern in all retinal layers. On the other hand, a physiological pattern of these enzymes was actually lost upon KA treatment in animals that were not treated with the drug. 

With respect to high glucose, which is highly toxic for neuronal cells, citicoline infusion was reported to improve the viability of retinal cells and reduce the extent of fibers loss upon exposure to high glucose in tissue cultures of mouse retinal explant and in retinal cells that were isolated from Sprague–Dawley rats [30,31]. In both experimental models, citicoline treatment reduced the frequency of apoptotic figures (TUNEL staining) in RGCs population, a finding that was mirrored by the decrease of caspases activation. Furthermore, citicoline restored significant levels of the neurotrophic factor and regenerating neurites. 

Citicoline was further found to revert in rat primary retinal neurons the synaptic loss induced by the glucose challenge [27], likely because of a boosting of the neuroprotective role that is exerted by Muller glia cells through the downregulation of the ERK1-ERK2 pathway [32]. Further, neuropharmacological studies have described the neuroprotective and antiapoptotic activities of CDP-choline and of citicoline in rat models of cerebral ischemia through Mitogen Activated Protein Kinase (MAPK) pathways, in particular ERK1-ERK2; therefore, a more detailed molecular investigation on the citicoline-mediated regulation of this central metabolic pathway seems to be required [30,33].

Additional approaches strengthened the protective role of citicoline, which was administered in liposomal formulation, against high glucose in diabetic mice in vivo [34]. In this setting, citicoline opposed the pathological downregulation of synaptophysin in whole retinas, restoring its anti-inflammatory properties (by means of preventing the upregulation of NF-κB and TNF-α (Tumor Necrosis Factor α)). The drug further decreased the extent of retinal reactive gliosis, and it lowered the frequency of apoptotic figures in all retinal elements, such as photoreceptors, bipolar cells, and RGCs [35]. Moreover, the administration of citicoline in eye drops formulation (2%) improved the reduction of NFL and choroidal thickness, as from OCT investigations, in a mouse model of long-lasting type I diabetes [36]. Specifically, the citicoline effect almost nullified the inverse correlation between the mean glucose level that was monitored in the blood of the animals and progression in ill animals of the nerve fiber layer thickness, in particular of the retinal nerve fiber layer (RNFL) and inner plexiform layer (IPL).

Another stimulating pathway that was shown to be modulated by citicoline is that of sirtuins, a class III histone deacetylases, which plays a multifaceted role in controlling cell energy metabolism, coupling NAD+ sensing with transcription regulation, and, thus, are profoundly implicated in tuning neuroprotective pathways and neuroinflammation, among the others [25]. Citicoline in rat brains induced sirtuins expression, but also in cultured neurons and in lymphomonocytes, and observations in Sirt−/− rats suggested that a significant neurovascular protection that is conferred by citicoline (either directly or indirectly) is determined through SIRT-1 [37]. 

Similarly, the administration of citicoline, but not of choline or cytidine, was found to be protective in transient cerebral ischemia through the inhibition of phospholipase A2 (PLA2), which turned into a decreased tissue inflammation and redox imbalance [38], properties that were further confirmed in a rat model of ageing [39]. Interestingly, PLA2 hydrolyses cardiolipin, a major phospholipid of mitochondrial membranes, which assists in organelle structure, fission, metabolism, and energy production through oxidative phosphorylation, providing a rationale for the pro-homeostatic role of citicoline [40].

With respect to this last point, the stabilization of mitochondrial membranes, maintenance of energy metabolism, plasma and axons structural and functional integrity, as well as resistance to apoptosis by citicoline are expected to be further achieved through the promotion of sphingomyelin and cardiolipin biosynthesis [15]. Furthermore, it is worth recalling that the stabilization of the plasma membrane through the increased bioavailability of phospholipid precursors helps in preventing glutamate release and toxicity.

In additional experimental models of retina degeneration following optic nerve crush, citicoline administration was associated with an improved viability and delayed degeneration of RGCs through decreased Bcl-2 levels, a pro-apoptotic protein; similarly, pre-treatment with the drug prevented the glutamate excitotoxicity death of cerebellar granule cells in vitro [41,42].

A relevant pharmacological and molecular hint to explain citicoline biological activity comes from research on the family of sigma-1 receptor, a chaperone-protein that is located in the Endoplasmic Reticulum (ER), where it plays multifaceted biological functions, including the regulation of Ca^2+^ flux and activation of neuroprotective pathways, such as autophagy [43,44]. 

In this framework, choline, which also acts as an intracellular messenger, is an endogenous agonist of sigma-1, providing a molecular link to the activity of this receptor with cholinergic synaptic activity [43]. 

In the eye, a pioneer study reported the sigma-1 receptor to be highly expressed in the rabbit iris-ciliary body. Indeed, pharmacological modulation of the receptor(s) with the topical administration of selected ligands was shown to modulate retinal protection and intraocular pressure [44]. Furthermore, sigma-1 receptor was found to confer protection to retinal pigment epithelium cells against oxidative stress, improving cell survival and DNA damage, clearly indicating that the modulation of this protein is also an attractive target in ophthalmology disorders as well as in neuro-degenerative disorders [45].

Hence, these studies depict a scenario where citicoline exerts its multifaceted neuroprotective activity by regulating sophisticated metabolic pathways at both the transcriptional and post-synthetic level, envisaging that the drug binds multiple targets, standing at the crossroad of cell metabolism. 

Furthermore, the known chemical composition makes it a very versatile molecule and, from a purely biochemical point of view, besides fueling the phospholipid bio-synthetic pathways that are mentioned above, the nucleotide and choline moieties (either alone or phosphorylated and joined together to form the intact citicoline) are particularly suited to dock into the functional or catalytic sites of biological macromolecules through non-covalent interactions. In the case of proteins, it is conceivable that citicoline may modulate their biological and/or enzymological properties, as it is supposed to do with PLA2 for instance. 

In this framework, the neuroprotection that is conferred by citicoline may come from those pathways that best cope with the toxic insults post mitotic cells are particularly vulnerable to, such as redox unbalance, proteotoxicity, and alteration of the proteostasis network (PN) [46,47]. PN is a term that defines the equilibrium between protein synthesis and degradation that every cell has to keep balanced to maintain viability, morphological, functional properties, and the fitness of the tissue it belongs to. Nevertheless, the choline-mediate activation of sigma-1 receptor and the downstream effect of this pathway fits with the PN improvement well.

Neurons, as well as other post-mitotic cells, are particularly vulnerable to this kind of insult, since they experience a very accelerated metabolic activity and, at the same time, have not the possibility to dilute the wasting by-products through cell mitosis [46,47]. A paradigmatic example of a PN unbalance, in which the accumulation overrides the bulk degradative capacity, is the deposition of amyloidogenic proteins (e.g., Aβ peptide in AD, α-synuclein in PD, among the others) in the intracellular and extracellular compartments during the onset and progression of neurodegenerative diseases [48,49].

Under homeostatic conditions, the early accumulation of proteotoxic species is balanced by the hyperstimulation of catabolic pathways that survey the proteome expressed at any given time by the cell. 

These catabolic pathways are largely known as intracellular proteolytic pathways, e.g., the Ubiquitin Proteasome System (UPS) and autophagy, which are more extensively reviewed elsewhere [50,51,52,53,54,55,56,57,58,59].

A recent research by our group cast light on a high affinity interaction of citicoline with the proteasome, a multi-subunit enzymatic assembly that carries out the regulated proteolysis of proteins, usually short-lived, tagged with a poly-ubiquitin chain [56]. 

**Table 1:** Summary of pre-clinical studies evaluating the therapeutic efficacy and the mechanism of action of Citicoline in vitro and in vivo.

Authors	Year	Study Design	Experimental Model	Insult	Citicoline Dose	Main Results
Martinet M. et al. [13]	1979	Case-Control	Rats	N/A ^a^	50 mg/Kg^−1^ (i.p.)	Dopamine and tyrosine synthesis in corpus striatum
Giménez R. et al. [40]	1998	Comparative Study	Sprague–Dawley Rats	N/A ^a^	100–500 mg/Kg (food intake)	PFA ^b^ accumulation with age in brain striatum
Alvarez X.A. et al. [57]	1999	Case-Control	Sprague–Dawley Rats	Hippocampal amyloid beta injection and permanent unilateral occlusion of carotid artery	62.5–250 mg/Kg^−1^ (i.p.)	Neuroprotection from A beta induced degeneration and hypoperfusion
Krupinsky J. et al. [37]	2002	Case-Control	Sprague–Dawley Rats	Middle Cerebral Artery Occlusion	500 mg/Kg^−1^ (i.p.)	Apoptosis rate in retina layers
Rejdak R. et al. [12]	2002	Case-Control	Albino Rabbits	N/A ^a^	50 mg/Kg (i.p.)	Retinal catecholamine levels
Oshitari T. et al. [30]	2002	N/A ^a^	Tissue Culture of Murine Retinal Explant	High Glucose	0.1–1 µM	Determination of apoptosis rate in RGCs ^c^; neurite proliferation
Adibhatla R.M. et al. [39]	2003			Transient Cerebral Ischemia		Effect on PLA2 ^d^ activity
Mir C. et al. [46]	2003	N/A ^a^	Cerebellar Granule Cells	Glutamate Toxicity	100 µM	Apoptosis rate
Krupinsky J. et al. [35]	2005	Case-Control	Sprague–Dawley Rats	Middle Cerebral Artery Occlusion	500 mg/Kg^−1^ (i.p.)	Activation of MAP ^e^ kinase (ERK-1/2) in retinal layers
Park C.H. et al. [29]	2005	Case-Control	Sprague–Dawley Rats	Kainic Acid (KA)	500 mg/Kg^−1^ (i.p.)	Thickness of retinal layers; IHC ^f^ staining of ChAT ^g^ and TH ^h^
Schutteauf F. et al. [42]	2006	Case-Control	Brown Norway Rats	Optic Nerve Crush	0.5 mg–2 g/Kg (i.p.)	RGCs ^c^ density; Bcl-2 IHC ^f^
Hurtado O. et al. [28]	2008	Comparative Study	Fischer Rats	Middle Cerebral Artery Occlusion	2 g/Kg^−1^ (i.p.)	Association of EAAT2 with lipid rafts in rat brain; glutamate uptake
Schauss A.G. et al. [24]	2009	Toxicol. Study	Sprague–Dawley Rats	N/A ^a^	Up to 2000 mg/Kg (oral gavage)	Mortality; blood and renal parameters assessment
Oshitari T. et al. [31]	2010	N/A ^a^	Retinal Cultures of Sprague–Dawley Rats	High Glucose	1 µM	Apoptosis rate in RGCs ^c^ population (TUNEL assay), caspases activation; regenerating neurites
Hurtado O. et al. [38]	2013	N/A ^a^	Fischer Rats wild-type and Sirt1^-/-^	Permanent Focal Ischemia	0.2–2 g/Kg^−1^(i.p.)	Citicoline efficacy in the absence of SIRT1
Matteucci A. et al. [27]	2014	N/A ^a^	Wistar Rat Embryos	Excitotoxicity and High Glucose	10–1000 µM	Apoptosis rate; caspases activation
Maestroni S. et al. [36]	2015	Comparative Study	C57Bl/6 Mice	Type I Diabetes	2% Citicoline-Ophthalmic Solution	Improvement of retinal layers thickness, in particular of RNFL ^i^ and IPL ^l^
Bogdanov P. et al. [34]	2018		Db/db Mouse	Obesity-Induced Type II Diabetes	2% Citicoline-Ophthalmic Solution	Apoptosis rate; glial activation; proinflammatory pathways activation; synaptophysin IHC ^f^
Carnevale C. et al. [26]	2019	Case-Control	Human	N/A ^a^	2% Citicoline-Ophthalmic Solution	Citicoline concentration in humor vitreous
Sbardella D. et al. [56]	2020	N/A ^a^	Biochemical assay and Human Neuron-derived Cell Lines	N/A ^a^	1–100 µM	Allosteric Modulation of proteasome; increased UPS ^m^ activity in cultured cell lines

^a^ N/A = Not Applicable; ^b^ PAF = Platelet Activated Factor; ^c^ RGCs = Retinal Ganglion Cells; ^d^ PLA2 = Phospholipase A2; ^e^ MAP = Mitogen Activated Protein; ^f^ dIHC = Immunohistochemitry; ^g^ ChAT = Chatecolamine Acetyl Transferase; ^h^ TH = Tyrosine Hydroxylase; ^i^ RNFL = Retinal Nerve Fiber Layer; ^l^ IPL = inner plexiform layer; ^m^ UPS: Ubiquitin Proteasome System.

Citicoline was found to significantly enhance, through a purely allosteric phenomenon, the proteolytic activity of the chymotrypsin-like activity either on synthetic substrates and/or on an amyloidogenic protein, such as α-synuclein. In this regard, it is worth pointing out that citicoline was found to milden the accumulation of implanted Aβ peptide in the hippocampus of Sprague–Dawley rats, improving the overall survival of neurons [57]. Aβ peptide is a proteasome substrate and, notably, is a recognized substrate of enzymes that physiologically interact with this multi-subunit complex, such as Insulin Degrading Enzyme and Neprylisin, which may become an interesting matter of investigation to search out for new biological activities of citicoline [50,51,52].

Furthermore, citicoline administration to neuron-derived human cells was found to stimulate the clearance of intracellular natural substrates of the UPS (e.g., poly-ubiquitinated proteins) and induce a structural rearrangement of the proteasome particles (increased association of 20S and 19S to form the 26S, see [56]), which maximized the proteolytic burden of the whole UPS over time.

This last point deserves some additional comments. The authors of this review firmly believe that citicoline stimulation of proteasome, if confirmed to occur also in vivo, is likely among the multifaceted ways through which the drug carries out neuroprotection. In this framework, the complex, hierarchical, and chronologically-tuned organization of the UPS provides a solid molecular basis to further explore metabolic pathways that are closely intertwined with it. The proteasome structural modulation documented can be interpreted into two non-mutually exclusive ways, namely: (a) citicoline directly induces a structural phenomenon that stimulates the association of the two proteasome subcomplexes (e.g., 20S and 19S) by increasing the binding affinity and (b) in the presence of citicoline, one or more of those pathways that finely tune proteasome composition gets activated and proteasome modulation is an indirect consequence of upstream events induced by citicoline. Among those reported to affect proteasome activity, it is worth mentioning the Calcium2+-dependent Calmodulin Kinase pathways (CamKII) and Protein Kinase A (PKA), which have a profound role in neuroprotection and post-mitotic cells metabolism [59,60]. 

Therefore, in order to fully characterize the biology of citicoline and to pave the road to further unexplored strategies of intervention based on its usage in clinics, it looks relevant to get clues, from single molecular observation, that allow for working backwards, digging inside the complexity of the intracellular metabolic pathways this small molecule likely joins in vitro and in vivo.

## 3. Clinical Data

Table 2, Table 3 and Table 4 summarize the psychophysical, morphological, and electrophysiological evidences that were obtained by citicoline treatment in OAG, AION, and DR patients.

### 3.1. Open Angle Glaucoma

Glaucoma is a neurodegenerative disease that is characterized by progressive retinal ganglion cells death. It is one of the most common worldwide causes of irreversible vision loss and its prevalence is increasing, since it is directly related to aging [61]. Quigley and Broman estimated that in 2020 there were almost 80 million people suffering from glaucoma and more than 10 million people bilaterally blind from glaucomatous optic neuropathy [62]. The first line therapy in the attempt to stabilize, or at least minimize, the damage to optic nerve head is still based on intraocular pressure (IOP) lowering strategies, but there are cases in which, despite a normal or even low IOP, damage progression can occur [63]. A lot of substances have been proposed to have the capacity to contrast RGCs degeneration in the last decades and citicoline seems to be one of the most promising [64]. 

Many studies tried to provide evidence that citicoline can be beneficial for glaucoma patients and, from these trials on visual field, visual pathways, and retinal nerve fiber layer in glaucoma patients (Table 2, Table 3 and Table 4), it emerged that citicoline, administered in addition to the ocular hypotensive therapy, brought about beneficial effects. 

#### 3.1.1. Psychophysical Evidences

The first study investigating the clinical benefits of treatment with citicoline on OAG, dates back to 1989, when Pecori Giraldi et al. [65] obtained the first evidence that citicoline can ameliorate the visual field defects in patients with well controlled IOP. In this study, forty-seven eyes from 30 patients suffering from OAG with typical visual field changes were treated with intramuscular administration of citicoline (1 g) for 10 consecutive days and examined at baseline, after 15 days and three months from the beginning of treatment, with central computerized perimetry (36 eyes), automated perimetry (11 eyes), and applanation tonometry. Despite the presence of many limitations of this study, it was very clear that citicoline could be considered to be a useful complement to the traditional IOP lowering therapies: the authors reported that 75% of the enrolled eyes had a significant reduction in the scotomatous areas with the central computerized perimetry or in the mean defect with the automated perimetry. In addition, beneficial effects could be maintained for at least three months and the improvement could be boosted if treatment was repeated, without clinically relevant side effects [65]. The same research group conducted another study to evaluate if the positive effects could last for years, with periodically repeated administration cycles. Unlike the first study, in this clinical trial, the enrolled patients were divided into two groups: eleven patients received an intramuscular administration of 1 g/day of citicoline for 15 days, repeated every six months, while twelve patients in the control group did not receive additional treatment. Patients were clinically tested for 10 years, with an applanation tonometry each month, a complete ophthalmologic examination every three months, and a Video Screen Perimetry every six months. Clinical worsening was defined as the increase of the non-perception area (NPA) on the visual field examination of at least 500 mm^2^ on at least two consecutive examinations. While, in the untreated group, a progressive NPA increase was found, the citicoline-treated patients had a significant reduction of NPA, with a long-lasting effect that consists in a significant improvement of retinal sensitivity that was confirmed during the next nine years [66]. Although the study groups were small, this was the first relevant evidence that the visual performance among glaucoma patients could be enhanced by a treatment with citicoline [60] and not only by hypotensive therapy [67]. Because people suffering from chronic ophthalmic disease do not usually accept long-term use of intramuscular injections well, further studies tried to find out different routes of administration. 

Ottobelli et al. provided more recent evidence of long-term benefits of citicoline in a prospective multicenter open clinical study [68]. In this before-after study, the OAG patients could be eligible if they had a retrospectively documented progression of at least −1 dB/year in mean deviation (MD) of Humphrey field analyzer 24-2 SITA Standard visual field (HFA 24-2) for at least three years, despite a good IOP control. During the two-year prospective phase of the study, the enrolled patients were treated with citicoline administered in oral solution (Neukron Ofta^®^, Omikron Italia, Rome, Italy), with the following scheme: one vial per day containing 500 mg of citicoline in oral solution for four months, followed by a two-month wash-out for which a bio-availability of approximately 98% of the oral solution (similar to that of endovenous administration) was previously documented [69,70]. Every patient repeated this cycle four times and a complete ophthalmologic examination and a HFA 24-2 were performed before each study treatment change (baseline plus month 4, 6, 10, 12, 16, 18, 22, and 24). At the end of the study, the mean rate of progression (RoP) passed from −1.1 dB/year to −0.15 dB/year, with a statistically significant difference: treated patients, with a previous fast progression, had only lost 0.3 dB of MD in two years. Despite the absence of a control group represents a big limitation of this study, the choice of enrolling fast progressing patients made the results interesting and worthy of further investigations [68]. 

By using a treatment scheme similar to Ottobelli et al. [68], Lanza et al. [71] evaluated 60 OAG patients with a RoP ranging from −1 to −1.5 dB/year in the last two years. In this study, OAG patients that were treated with citicoline administered in oral solution (Neukron Ofta^®^, Omikron Italia, Rome, Italy) showed a significant change of RoP that dropped to −0.45 db/year.

Since 2011 researchers’ attention moved in searching a formulation that permits the presence of citicoline in the eye posterior chamber and, therefore, with a potential action near those retinal elements located in the posterior segment that are involved in glaucoma (RGCs).

Thus, a formulation of citicoline by eye drops was developed that contained a solution of 2% of citicoline in addition to hyaluronic acid and benzalkonium chloride (OMK1^®^, Omikron Italia, Rome, Italy) or without benzalkonium chloride in Liposomal Formulation (OMK1-LF^®^, Omikron Italia, Rome, Italy). Two studies, performed in mice [72] and in humans [26], respectively, documented a significant concentration of citicoline and its metabolites in the vitreous chamber after eye drops administration. 

By using citicoline that was administered by eye drops (one drop three times/day), Roberti et al. [72] observed that, in OAG patients with an MD up to 12 dB, after two months of treatment, followed by one month of wash-out, the values of HFA 24-2 MD and Pattern Standard Deviation (PSD) did not improve significantly with respect to baseline ones. 

It is interesting to report the results of the study conducted by Parisi et al. [73], wherefore, despite a very small population, an MD improvement (defined as a change of at least 1 dB from baseline) was observed in 17 out of 24 OAG eyes (70%) after four months of treatment with citicoline administered by eye drops (one drop three times/day).

More recently, a randomized clinical trial (prospective, multicentric, and placebo-controlled study) was performed using citicoline 2% eye drops (OMK1^®^) on OAG patients with an MD ranging from −2 to −15 dB [74]. In this study, 80 patients were enrolled with a mild RoP in the two years before the baseline visit (−0.77 dB/year), despite a good IOP control. Four visits per year for three years were planned: each visit included a complete ophthalmologic examination and an HFA 10-2 and 24-2 tests. After three years of treatment, OAG patients that were treated with citicoline 2% eye drops showed a slower RoP of both HFA 10-2 and 24-2 MD (−0.14 dB/year and 0.34 dB/year, respectively), as compared to that observed in OAG patients that were treated with placebo (−0.74 dB/year and −0.64 dB/year, respectively).

#### 3.1.2. Morphological Evidences 

In addition to psychophysical evidence, the studies conducted by Lanza et al. [71] and Rossetti et al. [74] also evaluated the RNFL thinning on optic nerve head by Optical Coherence Tomography (OCT) examination. 

In the first study [71] after 12-months of follow-up, patients that were treated with citicoline administered in oral solution (Neukron Ofta^®^, Omikron Italia, Rome, Italy) showed a significantly slower RNFL thinning, as compared to control group and this difference appeared to be stable in the following 12 months. In nasal and temporal sectors, differences in the RNFL thickness between the two groups were relevant starting at sixmonths follow-up, with higher values in patients that were treated with citicoline. Differences in superior and inferior sectors were significant after 12 months of treatment. 

The randomized clinical trial, which was conducted by Rossetti et al. [74], highlighted similar results: patients that were treated with citicoline eye drops (OMK1^®^, Omikron Italia, Rome, Italy) showed a slower overall RNFL thinning, as compared to placebo group, but, in contrast to the previous study, the difference became statistically significant starting at 30-monhts follow-up. In a more detailed analysis, only inferior sectors showed relevant differences between the two groups. Patients that were treated with citicoline showed a 38% of reduction of RNFL thinning, with −0.62 micron/year versus −1 micron/year of the placebo group. Thus, the significant association between 10-2 MD changes and RNFL changes suggests that the RoP is influenced by the reduction of the RNFL progressive loss.

At present, studies aiming to compare the reduction of the RNFL progressive loss in OAG patients (homogenous for MD, age and RoP) treated either with eyes drops or systemic solutions/injections containing citicoline are not available. Therefore, actually, it is not possible to define whether the effect on RNFL thickness could be differently induced by treatment with citicoline by eye drops or by systemic (oral solution) administration. 

#### 3.1.3. Electrofunctional Evidences

Different electrophysiological methodologies allow for selectively evaluating RGCs function and the neural conduction along visual pathways. 

In particular, the peak-to-peak amplitude between the P50 and N95 components (P50-N95 amplitude) of the Pattern Electroretinogram (PERG) is the electrophysiological index that describes the functional integrity of the innermost retinal layers (RGCs and their fibers) [75,76]. By recording Visual Evoked Potential (VEP) and using appropriate visual stimuli (i.e., checkboards subtending either 15 or 60 min. of visual arc), it is possible to obtain bioelectrical responses of the visual cortex that describe the condition of the neural conduction along the small and large axons forming the visual pathways. The main parameter of VEPs responses is the implicit time of the P100 component (P100 implicit time) and the peak-to-peak amplitude between the N75 and P100 components (N75-P100 amplitude) [77,78]. In addition, by using simultaneous VEP and PERG recordings, it is possible to derive the electrophysiological index of the post-retinal neural conduction, which is called Retinocortical Time (RCT), calculated by the difference between VEP P100 and PERG P50 implicit times [79].

The first work was published in 1999, in which changes of PERG and VEP responses were observed in relation with the treatment with citicoline [80]. In this study, 40 OAG patients were enrolled with MD between −3 and −6 dB. They were randomly divided into two groups: 25 OAG patients were treated with citicoline (GC Group) and 15 patients were treated with placebo (GP Group). The GC patients were treated with citicoline that was administered intramuscularly (1000 mg/day, Neuroton^®^, Nuovo Consorzio Sanitario, Rome, Italy) fir 60 days. GP patients were treated with placebo for 60 days. At day 180 (after two months of wash-out), GC patients were sub-grouped: in 10 patients (GC1 group) the washout was prolonged for further 120 days; in 15 patients (GC2 group), a second treatment with intramuscular citicoline was administered for 60 days, followed by 120 days of washout. By contrast, in GP patients, at day 180, another period of washout was proposed for additional 180 days. 

GP patients displayed similar values of PERG and VEP parameters and of RCT in all examinations performed. In GC patients, the treatment with citicoline induced a significant improvement of PERG and VEP parameters and of RCT values. After washout, in GC patients, although there was a worsening trend, the values of PERG and VEP parameters and of RCT were still improved. After a second period of washout, GC1 patients showed PERG and VEP and RCT values that were similar to baseline ones and to those of GP patients; by contrast, in GC2 patients, a second period of citicoline treatment induced a further improvement of the values of PERG and VEP parameters and of RCT.

This work was very relevant, because it suggested that citicoline may induce a real effect of neuroenhancement [2] on RGCs and on axons forming the visual pathways; this contrasted with the idea that the primary observed visual filed changes in OAG after treatment with citicoline [66] could be related to an increase of the consciousness level [74,81] with a better performance during the execution of the psychophysical test. 

In a part of the patients (12 GC and 12 GP) enrolled in this study [80], the follow up was extended to a total period of 96 months [82]. In GC patients, a second treatment with intramuscular citicoline of two months was performed for 16 periods in eight years. Each treatment segment was followed by four months of wash-out. In the following periods (13–96 months) of citicoline administration, a great improvement of VEP and PERG values as well as RCT index was observed in GC patients, when comparing the data to the pre-treatment condition and to those observed in GP patients.

In addition, during the same period of follow-up, a worsening of the visual field defects was detected in GP patients, whereas, in the GC group, MD stabilization or improvement was found and the MD changes were significantly related to the increase of PERG P50-N95 amplitudes and to the shortening of both VEP P100 implicit times and RCTs.

In 2003, Rejdak et al. [83] suggested the administration of citicoline by oral route (one tablet containing 1000 mg of citicoline/day for 56 days) in OAG, and they observed a shortening of VEPs P100 implicit time in 62% of treated patients. 

In 2008, Parisi et al. [84] published a work involving OAG patients with an MD between −2 and −14 dB, where the effects on PERG and VEP responses of the intramuscular treatment with citicoline (1000 mg/day intramuscularly, Cebroton^®^, Tubilux Pharma, Pomezia, Rome, Italy) were compared to those that were related to a treatment performed administrating citicoline in oral suspension (1600 mg/day, Cebrolux^®^, Tubilux Pharma, Pomezia, Rome, Italy) for two sequential periods of two months of treatment (each followed by four months of wash-out) during a total period of 12 months of follow-up. The main result of this study was that the changes of the values of all electrophysiological parameters (i.e., PERG P50 or VEP P100 implicit times, PERG P50-N95 amplitudes, and RCT) were not statistically different between the two different types of citicoline administration.

The possible improvement of RGCs function and the neural conduction has been also evaluated when citicoline was available by eye drop administration.

In 2014 [72], 2015 [73], and 2019 [85], studies describing treatment with citicoline administered by eye drops with benzalkonium chloride (OMK1^®^, Omikron Italia, Rome, Italy) or preservative free formulation with a Liposomal System (OMK1-LF^®^, Omikron Italia, Rome, Italy) as carrier for citicoline were published. It was reported that citicoline eye drops induces an improvement of the PERG P50-N95 amplitudes and a shortening of VEPs P100 implicit times in OAG patients with an MD between −2 and −6 dB, either treated for a period of two [72] or four [73,85] months. It was interesting to observe that, when OAG patients were treated with OMK1^®^ [73], the improvement of RGCs function (detected by an increase of PERG P50-N95 amplitude) and neural conduction along the visual pathways (detectable from the shortening of the VEPs P100 implicit time) induced an improvement of HFA 24-2 MD with a RoP of 0.56 dB; by contrast, in OAG patients, which were treated exclusively with hypotensive drops, the RoP was of −0.24 dB.

### 3.2. Anterior Ischemic Optic Neuropathy

AION is the most common type of acute optic neuropathy in subjects that were aged more than 50 years, due to a deficit of blood supply to the optic nerve from the posterior ciliary arteries [86,87]. It represents an ophthalmological emergency, which is characterized by sudden and severe visual loss, visual field deficit, peripapillary hemorrhages, and optic nerve head swelling. AION can be non-arteritic (NAION) or arteritic (AAION), with a different prognosis, commonly being NAION, a self-limiting process accompanied by a partial resolution of the visual loss, and leading AAION to a more detrimental impairment of vision up to blindness [87]. Controversies on pathogenesis, systemic and local risk factors, clinical features, and especially on the management of NAION are due to various misconceptions [88]. Regarding the pharmacological management, it is generally accepted that systemic steroid therapy during early stages of NAION has a significant beneficial effect for visual outcome. 

Emerging neuroprotective treatments have been reported to counteract secondary neurodegeneration of RGCs and their axons [88]. Specifically, pivotal studies have described the efficacy of oral citicoline to enhance RGCs and visual pathway’s function (neuroenhancement) [89,90], as well as to induce the preservation of RGCs fibers (neuroprotection) [90]. The rationale of neuroprotection in NAION resides in the fact that not 100% of the axons forming the optic nerve die during an ischemic process, but only a portion of them is damaged. The surviving axons belonging to not damaged RGCs, which are responsible for the residual visual acuity and visual field sparing, are the targets of any protective strategy for subsequent improvement [88,91]. 

Here, we provide details on the effect on visual acuity and visual field, visual pathways and RGCs’ function, and RNFL structure in chronic NAION patients, after the administration of oral citicoline available since 2005 as an oral dietary supplement (Table 2, Table 3 and Table 4).

**Table 2:** Summary of clinical studies evaluating the effects of Citicoline in human ophthalmological neurodegenerative disease: electrophysiological evidences.

Authors	Year	Study Population	Administration	Dosage	Schedule of Treatment	Follow-up	Main Results
Parisi V. et al. [80]	1999	OAG ^a^ with MD ^g^ −3/−6 dB	IM ^b^	1000 mg/day	2 cycles of 60 days of treatment each followed by 120 days of wash-out	360 days	Increase in PERG ^c^ P50-N95 and in VEP ^d^ N75-P100 as ^e^ Shortening in PERG ^c^ P50 and VEP ^d^ P100 Its ^f^
Redjak R. et al. [83]	2003	OAG ^a^	Tablet	1000 mg/day	1 cycle of 56 day of treatment	56 days	Shortening in PERG ^c^ P50 and VEP ^d^ P100 Its ^f^
Parisi V. [82]	2005	OAG ^a^ with MD ^g^ −3/−6 dB	IM ^b^	1000 mg/day	14 cycles of 60 days of treatment each followed by 120 days of wash-out	8 years	Increase in PERG ^c^ P50-N95 and in VEP ^d^ N75-P100 as ^e^; Shortening in PERG ^c^ P50 and VEP ^d^ P100 Its ^f^ Correlation with Visual Field improvement.
Parisi V. et al. [84]	2008	OAG ^a^ with MD ^g^ −2/−14 dB	IM ^b^ Oral	1000 mg/day 1600 mg/day	2 cycles of 60 days of treatment each followed by 120 days of wash-out	360 days	Increase in PERG ^c^ P50-N95 and in VEP ^d^ N75-P100 as ^e^ Shortening in PERG ^c^ P50 and VEP ^d^ P100 Its ^f^ Non-significant differences between IM ^b^ and Oral treatment.
Parisi V. et al. [89]	2008	NAION ^h^	Oral Suspension	1600 mg/day	2 cycles of 60 days of treatment each followed by 120 days of wash-out	360 days	Increase in PERG ^c^ P50-N95 and in VEP ^d^ N75-P100 as ^e^ Shortening in PERG ^c^ P50 and VEP ^d^ P100 Its ^f^
Roberti G. et al. [72]	2014	OAG ^a^ with MD ^g^ −2/−14 dB	Eye drops	3 drops/day	60 days of treatment followed by 30 days of wash-out	90 days	Increase in PERG ^c^ P50-N95 as ^e^ Shortening in PERG ^c^ P50 Its ^f^ Association with Visual Field improvement.
Parisi V. et al. [73]	2015	OAG ^a^ with MD ^g^ > 12 dB	Eye drops	3 drops/day	120 days of treatment followed by 60 days of wash-out	180 days	Increase in PERG ^c^ P50 -N95 and in VEP ^d^ N75-P100 as ^e^ Shortening in PERG ^c^ P50 and VEP ^d^ P100 Its ^f^ Correlation with Visual Field improvement.
Parisi V. et al. [85]	2019	OAG ^a^ with MD ^g^ −2/−6 dB	Eye drops LF ^i^	3 drops/day	120 days of treatment	120 days	Increase in PERG ^c^ P50-N95 and in VEP ^d^ N75-P100 as ^e^ Shortening in PERG ^c^ P50 and VEP ^d^ P100 Its ^f^ Association with Visual Field improvement.
Parisi V. et al. [90]	2019	NAION ^h^	Oral Solution	500 mL/day	180 days of treatment followed by 90 days of wash-out	2700 days	Increase in PERG ^c^ P50-N95 and in VEP ^d^ N75-P100 as ^e^ Shortening in PERG ^c^ P50 and VEP ^d^ P100 Its ^f^ Association with Visual Field or RNFL-T ^l^ stabilization or improvement.

^a^ OAG = Open Angle Glaucoma; ^b^ IM = Intramuscular; ^c^ PERG = Pattern Electroretinogram; ^d^ VEP = Visual Evoked Potentials; ^e^ As = Amplitudes; ^f^ Its = Implicit Times; ^g^ MD = Mean Deviation of Humphrey field analyzer 24-2 SITA Standard strategy; ^h^ NAION= Non-arteritic Anterior Ischemic Optic Neuropathy; ^i^ LF = Liposomal Formulation; ^l^ RNFL-T= Retinal Nerve Fiber Layer Thickness.

#### 3.2.1. Psychophysical Evidences: Visual Acuity

After citicoline treatment in oral suspension (1600 mg/ day Cebrolux^®^, Tubilux Pharma, Pomezia, Rome, Italy), for two cycles of administration of 60 days each, interrupted by a wash-out period of four months, a significant increase in Visual Acuity (VA) in the 14 NAION treated eyes [89] was found. A reduction in VA was observed after the first wash-out period, though ameliorated with respect to the baseline values; over the same period, no significant VA changes were detected in the 12 not treated patients. After this initial steep increase, only a little progression of the VA at the end of the second two month treatment phase (180–240 days) was observed. After the second wash-out period, VA decreased, even though the values were still lower than the basal ones, maintaining the improvement in VA.

The effects of the treatment with citicoline administrated in oral solution (one vial of 500 mg daily, Neukron Ofta ^®^, Omikron Italia, Rome, Italy) on 19 NAION patients for six months, followed by three months of wash-out were also studied [90]. Significant individual changes for VA in the citicoline treated NAION patients were observed after six months and at nine months of evaluation.

#### 3.2.2. Psychophysical Evidences: Visual Field

In the latter study [90], the HFA 24-2 MD after treatment with citicoline administered in oral solution was also assessed. When comparing six and nine months to the baseline values, an MD improvement in 74% of treated patients was found. By contrast, an MD worsening was detected in 70% of 16 not treated NAION patients.

#### 3.2.3. Morphological Evidences

A morphological impairment of RNFL, characterized by initial swelling in the acute phase and by sectorial or diffuse thinning in the chronic phase of NAION, has been widely studied by using spectral domain (SD-OCT) [92]. More recently, it was also confirmed that, in chronic NAION, a selective impairment of the inner retina occurs, with outer retina sparing from neurodegeneration, as detected by segmenting the retinal layers that were scanned by SD-OCT [93].

When citicoline in oral solution was administered to chronic NAION patients for six months [90], SD-OCT RNFL thickness (RNFL-T) was used as the morphological index to describe the integrity of RGCs fibers forming the optic nerve. This was investigated to unveil the potential effect of neuroprotection on the optic nerve by exogenous citicoline. At baseline, both treated and not-treated NAION Groups showed a significant reduction in RNFL-T in all sectors as compared to Controls. This was likely due to loss of nerve fibers typical of the chronic NAION, and described to occur right after one or two months from the acute onset of visual loss [94,95,96].

After six months of treatment with citicoline in oral solution, a large percentage of eyes showed unmodified or improved RNFL overall thickness, with no worsening. By contrast, untreated NAION eyes showed a significant progressive thinning of the overall RNFL. These relevant results were attributed to the potential property of citicoline to limit RGCs and their fibers death [90], as suggested by the effect of citicoline in controlling neuronal apoptosis and inducing the regeneration of new-born RGCs neurites in experimental models [31,97]. 

#### 3.2.4. Electrofunctional Evidences

Because VA and visual field are psychophysical parameters, highly subjective and influenced by the suggested citicoline effect of increased level of consciousness and attention [81,98], it was considered that VA and visual field were not the best parameters that are able to describe the potential beneficial effect of citicoline for enhancing visual function. Therefore, in the clinical practice settings and reported studies [89,90], psychophysical data are always combined with more objective, not invasive electrophysiological data.

PERG P50-N95 amplitude, as well as VEPs P100 implicit time and N75-P100 amplitude, have been widely used in the clinical settings of NAION to assess RGCs dysfunction [89,99,100] and the impairment of neural conduction along the visual pathway [89,101,102], respectively. 

In a previous NAION study [89], the simultaneous PERG and VEP recordings were used to assess the effect of citicoline that was administered in oral suspension (Cebrolux^®^, Tubilux Pharma, Pomezia, Rome, Italy) on the whole visual pathways from the RGCs to the visual cortex, knowing that it was significantly impaired as compared to the Controls. At the end of the two periods of treatment with citicoline a significant enhancement (with respect to baseline and not treated NAION) of the RGCs function was found, as suggested by greater PERG P50-N95 amplitude and by the reduction in PERG P50 implicit time in NAION treated patients. 

The improvement of RGCs function was accompanied by an enhancement of the function of the visual pathways, as demonstrated by ameliorated VEPs parameters (shortening of VEP P100 implicit times and improved N75-P100 amplitudes) and by better values of VA. After washout, the improvement of PERG values persisted as compared to the baseline condition. Therefore, unlike VA, which first improved and then stabilized, PERG and VEP improvement steadily progressed throughout the treatment period, only in NAION treated patients.

In a more recent study on the administration of citicoline in oral solution (Neukron Ofta ^®^, Omikron Italia, Rome, Italy) to NAION patients [90], a significant positive change in individual PERG P50-N95 amplitude, VEP P100 implicit time, and VEP N75-P100 amplitude at six months follow-up was found, as compared to the baseline; the VEP P100 implicit time shortening was not significantly correlated with relative changes in PERG amplitude. In contrast, in not treated NAION patients, no significant VEP changes were observed, while a significant reduction in PERG P50-N95 amplitude was detected by comparing the data to baseline values. In this pivotal study [90], the lack of a significant correlation between electrophysiological (PERG and VEP values) and morphological (RNFL-T values) data was also observed, and this suggests that citicoline administered in oral solution has concomitant, but unrelated, functional and structural effects on RGCs axons.

### 3.3. Diabetic Retinopathy

DR represents one of the most important complications of diabetes and preventable causes of visual impairment among people of working age and industrialized countries [103,104]. A high prevalence of DR of up to 86% in patients with type 1 diabetes mellitus (DM1) in Europe, south Asia, and the USA has been reported [105,106]. Genetic factors, the duration of the disease, poor glycemic control and variability, hyperlipidemia, and hypertension are considered to be important risk factors for the development and progression of DR [107,108,109,110,111].

Although DR has been always been considered to be a microvascular disease, in the early stages a degeneration of neural retinal elements, such as RGCs, has been demonstrated and it could be implicated in the development of the microvascular damage [112,113,114,115].

The retinal neurodegeneration could be induced by several factors, such as the increase of excitoxic metabolites (e.g., glutamate, branched chain amino acids, and homocysteine), the decrease of folic acid and vitamin B12 [116] or the activation of inflammasome by P2X7 receptor [117,118,119].

Many data support the role of the Citicoline on RGC by increasing its function (neuroenhancement) and preventing the neurodegeneration (neuroprotection) (see, as a review, Faiq et al. [15]). Recently, the effects of citicoline and vitamin B12 administered in eye drops on function, structure, and vascular condition of central retina in type 1 patients with mild signs of DR during a period of 36 months in comparison with placebo have been reported [120].

In this study, twenty patients were enrolled with DM1 and mild signs of non-proliferative diabetic retinopathy (NPDR); they were randomly divided into two groups, namely (a) the DC group, including patients that were treated with citicoline and vitamin B12 eye drops, and (b) the DP group, including those treated with placebo. In the DC group, one eye of each patient was treated with citicoline and vitamin B12 eye drops (three drops/day, OMK2^®^, Omikron Italia, Rome, Italy), while, in the DP group, it was treated with placebo (three drops/day, eye drops containing hypromellose 0.3%) for 36 months. In both of the groups, Humphrey Matrix frequency doubling technology (FDT), SD-OCT and OCT angiography (OCTA), and adaptive optics (AO) were performed at baseline, and 12, 24, and 36 months of the follow-up period.

**Table 3:** Summary of clinical studies evaluating the effects of citicoline in human ophthalmological neurodegenerative disease: psychophysical evidences.

Authors	Year	Study Population	Administration	Dosage	Schedule of Treatment	Follow-up	Main Results
Pecori Giraldi J. et al. [65]	1989	OAG ^a^	IM ^b^	1000 mg/day	10 days	90 days	Visual Field improvement and stability
Virno M. et al. [66]	2000	OAG ^a^	IM ^b^	1000 mg/day	20 cycles of 15 days of treatment each followed by 180 days of wash-out	10 years	Visual Field improvement
Parisi V. et al. [82]	2005	OAG ^a^ with MD ^g^ −3/−6 dB	IM ^b^	1000 mg/day	14 cycles of 60 days of treatment each followed by 120 days of wash-out	8 years	Visual Field improvement correlated with: Increase in PERG ^c^ P50-N95 and in VEP ^d^ N75-P100 as ^e^; Shortening in PERG ^c^ P50 and VEP ^d^ P100 Its ^f^
Parisi V. et al. [89]	2008	NAION ^h^	Oral Suspension	1600 mg/day	2 cycles of 60 days of treatment each followed by 120 days of wash-out	360 days	Increase in PERG ^c^ P50-N95 and in VEP ^d^ N75-P100 as ^e^ Shortening in PERG ^c^ P50 and VEP ^d^ P100 Its ^f^
Ottobelli L. et al. [68]	2013	Progressive OAG ^a^	Oral Solution	500 mg/day	4 cycles of 120 days of treatment each followed by 60 days of washout	2 years	Reduction of Visual Field rate of progression
Roberti G. et al. [72]	2014	OAG ^a^ with MD ^g^ −2/−14 dB	Eye drops	3 drops/day	60 days of treatment followed by 30 days of wash-out	90 days	Visual Field improvement associated to: Increase in PERG ^c^ P50-N95 as ^e^ Shortening in PERG ^c^ P50 Its ^f^
Parisi V. et al. [73]	2015	OAG ^a^ with MD ^g^ > 12 dB	Eye drops	3 drops/day	120 days of treatment followed by 60 days of wash-out	180 days	Visual Field improvement correlated with: Increase in PERG ^c^ P50-N95 and in VEP ^d^ N75-P100 as ^e^ Shortening in PERG ^c^ P50 and VEP ^d^ P100 Its ^f^
Parisi V. et al. [85]	2019	OAG ^a^ with MD ^g^ −2/−6 dB	Eye drops LF ^i^	3 drops/day	120 days of treatment	120 days	Visual Field improvement associated to: Increase in PERG ^c^ P50-N95 and in VEP ^d^ N75-P100 as ^e^ Shortening in PERG ^c^ P50 and VEP ^d^ P100 Its ^f^
Parisi V. et al. [90]	2019	NAION ^h^	Oral Solution	500 mL/day	180 days of treatment followed by 90 days of wash-out	270 days	Visual Field improvement associated to: Increase in PERG ^c^ P50-N95 and in VEP ^d^ N75-P100 as ^e^ Shortening in PERG ^c^ P50 and VEP ^d^ P100 Its ^f^
Lanza M. et al. [71]	2019	OAG ^a^	Oral Solution	500 mg/day	2 cycles of 120 days of treatment each followed by 60 days of wash-out	730 days	Reduction of Visual Field rate of progression Association with slower RNFL-T ^l^ and GCC-T ^m^ thinning
Rossetti L. et al. [74]	2020	OAG ^a^ with MD ^d^ −2/−15 dB	Eye drops	3 drops/day	1095 days of treatment	1095 days	Reduction of Visual Field rate of progression Association with slower RNFL-T ^l^ thinning
Parravano M. et al. [120]	2020	NPDR ^n^	Eye drops	3 drops/day	1095 days of treatment	1095 days	Increase in FDT ^o^ 10-2 Macular Sensitivity, Association with IPL ^p^ and OPL ^q^ thickness stabilization Foveal vessel density at SCP ^r^ and DCP ^s^ stabilization at OCTA ^t^

^a^ OAG = Open Angle Glaucoma; ^b^ IM = Intramuscular; ^c^ PERG = Pattern Electroretinogram; ^d^ VEP = Visual Evoked Potentials; ^e^ As = Amplitudes; ^f^ Its = Implicit Times; ^g^ MD = Mean Deviation of Humphrey field analyzer 24-2 SITA Standard strategy; ^h^ NAION = Non-arteritic Anterior Ischemic Optic Neuropathy; ^i^ LF = Liposomal Formulation; ^l^ RNFL-T = Retinal Nerve Fiber Layer Thickness; ^m^ GCC-T = Ganglion Cell Complex Thickness; ^n^ NPDR = Non-proliferative Diabetic Retinopathy; ^o^ FDT = Frequency Doubling Technology; ^p^ IPL = Inner Plexiform Layer; ^q^ OPL= Outer Plexiform Layer; ^r^ SCP = Superficial Capillary Plexus; ^s^ DCP = Deep Capillary Plexus; ^t^ OCTA = Optical Coherence Tomography Angiography.

#### 3.3.1. Psychophysical Evidences

By looking at the results in details, in the long term, Humphrey Matrix FDT 24-2 mean sensitivity (MS), PSD, and MD between the DC and DP groups appeared to be similar, whereas a significant difference of FDT 10-2 MS was detected between the two groups. In particular, a significant decrease of 10-2 MS in the DP group over time was found in comparison with the DC group, where no significant changes were observed during the follow up [120]. The reduction of MS may be related to both a higher susceptibility of the magno-cellular RGCs to hyperglycemia or lower redundancy of those cells among the entire RGCs, as already reported by Parravano et al., who reported an early functional impairment of RGCs through FDT in the eyes of the patients with DM1 [121].

#### 3.3.2. Morphological Evidences

By analyzing the morphological results, OCT parafoveal Inner Nuclear Layer (INL) thickness showed a significant increase the DP group over time in comparison with the DC group, where no significant changes were noted. Moreover, the Outer Plexiform Layer (OPL) thickness showed a significant reduction at 12, 24, and 36 months in the DP group, while the OPL values were similar over the entire follow up in the DC group [120]. A specific change of the retinal layers in DR, with the progressive increase in INL and decrease in OPL thickness, could indicate the activation of the glia and the Muller cells and the induction of subclinical macular edema, as early biomarkers of the development of the disease [122,123]. The microvascular evaluation that was obtained with OCTA showed that the foveal vessel density (VD) at superficial capillary plexus (SCP) and that at deep capillary plexus (DCP) were different between the DC and DP groups; in particular, the foveal VD at both SCP and DCP appeared to be reduced over time in the DP group in comparison with DC group, where no significant changes were observed. In addition, the area of the foveal avascular zone (FAZ) showed no significant changes over time in both groups. As already reported, the impairment of DCP represents a biomarker for both early diagnosis and risk of progression in DR [124].

With regard to the AO findings, the cone density, linear dispersion index, and heterogeneity packing index showed no significant changes between the DC and DP groups over time. In diabetic patients with DR, an interesting model to evaluate the neurodegenerative process is to assess the corneal innervation by means of in vivo confocal biomicroscopy of corneal sub-basal plexus [125]. A recent randomized clinical trial was performed on diabetic patients with DR randomized to receive citicoline and vitamin B12 eye drops (OMK2 ^®^, 3 drops/day) or placebo for 18 months, with the aim to evaluate the effect on corneal morphology (with confocal microscopy) and sensitivity (by corneal esthesiometry). In the group that was treated with citicoline, a significant increase of corneal nerve fiber length density and corneal sensitivity was detected with respect to those that were observed in the group treated with placebo [126]. 

**Table 4:** Summary of clinical studies evaluating the effects of Citicoline in human ophthalmological neurodegenerative disease: morphological evidences.

Authors	Year	Study Population	Administration	Dosage	Schedule of Treatment	Follow-up	Main Results
Lanza M. et al. [71]	2019	OAG ^a^	Oral Solution	500 mg/day	2 cycles of 120 days of treatment each followed by 60 days of wash-out	730 days	Slower RNFL-T ^b^ and GCC-T ^c^ thinning Association with visual field stabilization
Rossetti L. et al. [74]	2020	OAG ^a^ with MD ^d^ −2/−15 dB	Eye drops	3 drops/day	1095 days of treatment	1095 days	Slower RNFL-T ^b^ thinning Association with visual field stabilization (10-2)
Parisi V. et al. [90]	2019	NAION ^e^	Oral Solution	500 mL/day	180 days of treatment followed by 90 days of wash-out	270 days	RNFL-T ^b^ stabilization or improvement associated to: Increase in PERG ^f^ P50 -N95 and in VEP ^g^ N75-P100 as ^h^ Shortening in PERG P50 and VEP P100 Its ^i^ Improvement of Visual Filed
Parravano M. et al. [120]	2020	NPDR ^l^	Eye drops	3 drops/day	1095 days of treatment	1095 days	IPL ^m^ and OPL ^n^ thickness stabilization Foveal vessel density at SCP ^o^ and DCP ^p^ stabilization at OCTA ^q^
Fogagnolo P. el al. [126]	2020	DR ^r^	Eye drops	3 drops/day	540 days of treatment	540 days	Increase of corneal nerve fiber length density and of corneal sensitivity

^a^ OAG = Open Angle Glaucoma; ^b^ RNFL-T= Retinal Nerve Fiber Layer Thickness; ^c^ GCC-T = Ganglion Cell Complex Thickness; ^d^ MD = Mean Deviation of Humphrey field analyzer 24-2 SITA Standard strategy; ^e^ NAION= Non-arteritic Anterior Ischemic Optic Neuropathy; ^f^ PERG = Pattern Electroretinogram; ^g^ VEP = Visual Evoked Potentials; ^h^ As = Amplitudes; ^i^ Its = Implicit Times; ^l^ NPDR = Non-proliferative Diabetic Retinopathy; ^m^ IPL = Inner Plexiform Layer; ^n^ OPL= Outer Plexiform Layer; ^o^ SCP = Superficial Capillary Plexus; ^p^ DCP = Deep Capillary Plexus; ^q^ OCTA = Optical Coherence Tomography Angiography; ^r^ DR = Diabetic Retinopathy.

#### 3.3.3. Electrofunctional Evidences

At this moment, there is a lack of information regarding the possible effects of citicoline on the function of RGCs or of the retinal pre-ganglionic elements on patients with DM1 and mild signs of NPDR, as assessed by Patter-ERG and multifocal ERG, respectively.

## 4. Concluding Remarks

The rationale for the use of citicoline in ophthalmological neurodegenerative diseases is founded on its multifactorial mechanism of action and the involvement in several metabolic pathways including phospholipid homeostasis, mitochondrial dynamics as well as cholinergic and dopaminergic transmission, as reported in several pre-clinical studies. In addition, recent insights suggest an involvement of citicoline in the regulation of the intracellular proteostasis network through an interaction with the proteasome. 

Nevertheless, besides strong knowledge supporting its mechanisms of action, strong clinical evidences are required for an evidence-based clinical use of any compound. 

In addition to the evidences of an improvement of psychophysical, morphological, and electrophysiological outcomes, coming from small comparative randomized pilot clinical trials or larger not controlled before-after studies (performed in different ophthalmological neurodegenerative diseases, like OAG, AION, and DR), for the first time a reduction of the rate of visual field progression has been recently reported in OAG patients in a large randomized double-masked placebo-controlled clinical trial, thus further supporting the clinical use of citicoline. 

## Data Availability

No new data were created or analyzed in this study. Data sharing is not applicable to this article.

## Abbreviations

RGCs	retinal ganglion cells
ONH	optic nerve head
OAG	open angle glaucoma
AION	anterior ischemic optic neuropathy
DR	diabetic retinopathy
KA	kainic acid
MAPK	Mitogen Activated Protein Kinase
TNF-α	Tumor Necrosis Factor α
RNFL	retinal nerve fiber layer
IPL	inner plexiform layer
PLA2	phospholipase A2
PN	proteostasis network
UPS	ubiquitin proteasome system
CamKII	calmodulin kinase pathways
PKA	Protein Kinase A
IOP	intraocular pressure
NPA	non-perception area
HFA	Humphrey field analyzer
RoP	rate of progression
MD	mean deviation
PSD	pattern standard deviation
OCT	optical coherence tomography
PERG	pattern electroretinogram
VEP	visual evoked potential
RCT	retinocortical time
NAION	non-arteritic anterior ischemic optic neuropathy
AAION	arteritic anterior ischemic optic neuropathy
VA	Visual Acuity
Sd-OCT	spectral domain optical coherence tomography
RNFL-T	RNFL thickness
DM1	type 1 diabetes mellitus
NPDR	non-proliferative diabetic retinopathy
FDT	frequency doubling technology
OCTA	OCT angiography
AO	adaptive optics
MS	mean sensitivity
INL	inner nuclear layer
OPL	outer plexiform layer
VD	vessel density
SCP	superficial capillary plexus
DCP	deep capillary plexus
FAZ	foveal avascular zone
